# Machine Learning Algorithms for Predicting Mechanical Stiffness of Lattice Structure-Based Polymer Foam

**DOI:** 10.3390/ma16227173

**Published:** 2023-11-15

**Authors:** Mohammad Javad Hooshmand, Chowdhury Sakib-Uz-Zaman, Mohammad Abu Hasan Khondoker

**Affiliations:** Industrial Systems Engineering, Faculty of Engineering and Applied Science, University of Regina, Regina, SK S4S 0A2, Canada; mhb387@uregina.ca (M.J.H.); csy528@uregina.ca (C.S.-U.-Z.)

**Keywords:** polymer foams, lattice structures, mechanical stiffness, machine learning, additive manufacturing

## Abstract

Polymer foams are extensively utilized because of their superior mechanical and energy-absorbing capabilities; however, foam materials of consistent geometry are difficult to produce because of their random microstructure and stochastic nature. Alternatively, lattice structures provide greater design freedom to achieve desired material properties by replicating mesoscale unit cells. Such complex lattice structures can only be manufactured effectively by additive manufacturing or 3D printing. The mechanical properties of lattice parts are greatly influenced by the lattice parameters that define the lattice geometries. To study the effect of lattice parameters on the mechanical stiffness of lattice parts, 360 lattice parts were designed by varying five lattice parameters, namely, lattice type, cell length along the X, Y, and Z axes, and cell wall thickness. Computational analyses were performed by applying the same loading condition on these lattice parts and recording corresponding strain deformations. To effectively capture the correlation between these lattice parameters and parts’ stiffness, five machine learning (ML) algorithms were compared. These are Linear Regression (LR), Polynomial Regression (PR), Decision Tree (DT), Random Forest (RF), and Artificial Neural Network (ANN). Using evaluation metrics such as mean squared error (MSE), root mean squared error (RMSE), and mean absolute error (MAE), all ML algorithms exhibited significantly low prediction errors during the training and testing phases; however, the Taylor diagram demonstrated that ANN surpassed other algorithms, with a correlation coefficient of 0.93. That finding was further supported by the relative error box plot and by comparing actual vs. predicted values plots. This study revealed the accurate prediction of the mechanical stiffness of lattice parts for the desired set of lattice parameters.

## 1. Introduction

Polymer foams are used extensively for their mechanical properties, energy-absorption capabilities, low weight, exceptional cushioning qualities, and excellent insulating behavior [1,2]. Polymer foam can be defined as a two-phase system that consists of gas bubbles dispersed into a polymer matrix [3]. It has a wide range of application areas, including the automotive industry [4], engineering materials [5], packaging [6], thermal insulation [7,8], protection [9], housing decoration, mattresses, furniture, and electronic devices [10]. As polymer foams undergo large deformation under compression, understanding their mechanical behavior, especially deformation under different loading conditions, is crucial [10,11,12]. Functionally graded materials are inhomogeneous composites with modifiable features that are now employed extensively across a variety of industries [13]. Foam materials with varying degrees of functionality have been demonstrated to work well in shock-absorbing applications [14,15]. Recent studies have also asserted that these foam features can be significantly influenced by foam structure and morphology such as spatial distribution and gradient of cell size [16,17]. An asymmetric spatial feature can produce better mechanical and thermal-insulation outcomes, which can make them beneficial in a range of applications, such as impact resistance, high strength at low weight, and thermal or sound insulation [14,18]. It has been demonstrated that the use of functionally graded foam materials offers high performance in applications requiring compression resistance and shock absorption [14,15].

One important application of foam polymer is in the design of custom mattresses. As humans spend about one-third of their lives lying in bed, a custom mattress designed in accordance with body curvature and weight distribution is very important to relieve any back pain or discomfort [19]. While designing a mattress, three aspects are considered: the shape and mass distribution of the human body, the mechanical properties of the material of the mattress, and the interaction between the human body and the material [20,21]. Another prospective application is footwear due to the high impact load repetitively exerted on the feet, which is several times greater than body weight [22]. The use of proper footwear cushioning is necessary to prevent repetitive stress injuries since the high load is repeated during the walk [22,23]. Additionally, the right footwear can enhance exercise comfort and performance. Hence, with the right design of functionally graded foam materials, it is possible to create useful ergonomic items such as shoe soles. The soles of shoes should be lightweight and have adequate shock absorption and endurance [24,25]. Contemporary sports footwear is engineered to alter the viscoelastic midsole, which is commonly comprised of polymeric foam to reduce mechanical stress waves [26]. Similarly, athlete safety and the prevention of injuries are both crucial, which is why different foam constructions are employed for many areas in protective gear or for surfaces where sports activities can be practiced safely [2,22].

The drawback of foam materials, however, is that they are stochastic and have a random microstructure [27]. As the microstructure of these foam materials plays a crucial role in their global behavior and properties, researchers have tried to find predictable alternatives for foams [28,29]. Lattice structures, in particular, are the subject of substantial research due to their multi-functional properties, including load carrying [30], energy absorption [31], heat exchange [32], and building materials [33]. They are created by duplicating mesoscale unit cells in three dimensions. They offer extreme design freedom to alter the geometries of unit cells in order to attain desired macro-scale material attributes for a variety of applications [34]; however, producing these complex and intricate lattice structures can be infeasible using conventional manufacturing processes, in which case, the need for advanced manufacturing comes into play [35].

Additive manufacturing (AM), also known as 3D printing, is a cutting-edge technology that enables the production of complex geometries and near-net-shape components with minimal raw material consumption [36,37,38,39]. Utilizing the benefits of 3D-printing technology, functionally graded lattice materials can be manufactured with a uniform and ordered structure, and their unit cells can be manipulated and optimized to achieve the desired mechanical properties for a specific application [40,41]. Three-dimensional printed polymeric lattice structures have been studied for their uses in energy absorption [31], building materials [33], enhanced ductility [42], and mechanical properties [43]. 

A wide variety of factors can significantly impact the behavior of the 3D-printed lattice parts, which would, in turn, affect their mechanical behaviors. Therefore, understanding the relation between the lattice structural parameters and mechanical performance, such as stiffness, is of vital importance for the optimization of the lattice design [44]. In this context, machine learning (ML), a subset of artificial intelligence (AI), plays a vital role by analyzing the hidden links and patterns within a given dataset. ML uses data analysis to recognize patterns and connections, enabling it to perform specific functions. ML algorithms have a greater ability to detect non-linear interaction between the parameters of an AM process, and mechanical properties such as deformation, compared to conventional methods. AI- and ML-based tools play a crucial role in hastening the advancement of new materials, production methods, and processes [45]. The methods are divided into supervised learning, where the algorithm picks up knowledge from labeled training data and assists in making predictions for unforeseen data, and unsupervised learning, where the algorithm defines how to establish relationships between features of interest by working with unlabeled data [45]. Building connections and drawing conclusions from data, systems, or frameworks, with the ability to automatically learn and improve without explicit programming, can be facilitated using ML techniques [46]. In this study, a number of lattice structures were designed and computational analyses were performed to understand the effect of lattice geometries on their mechanical stiffness. Then, different ML algorithms were evaluated to study their performance.

## 2. Methodology

### 2.1. Data Generation

In this work, nTop (https://www.ntop.com/ (accessed on 13 November 2023), New York, NY, USA) was utilized with a non-commercial license to design a total of 360 lattice unit cells by changing five lattice parameters, namely, lattice type, cell length along the X, Y, and Z axes, and cell wall thickness. Once designed, these lattice structures were subjected to the same loading conditions using the nTop Simulation module. Then, corresponding strain deformations were recorded to form a dataset that was analyzed by ML algorithms to establish a correlation among them.

### 2.2. Designing Lattice Structures

**Figure 1:**
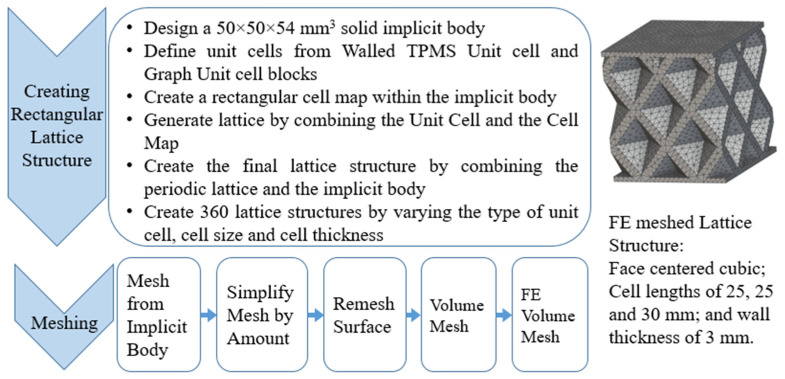
Flow chart of the design process.

Using the lattice parameters of unit cells listed in Table 1, lattice structures with a volume of 50 × 50 × 54 mm^3^ were designed in nTop by following the workflow outlined in Figure 1. nTop offers six walled triply periodic minimal surface or WTPMS-type unit cells and 29 graph-type unit cells, as presented in Table 2.

To design different lattice structures, a 50 × 50 × 50 mm^3^ cube was designed in nTop. Then, a 2 mm thick plate was added at the top and bottom surface of the cube using the “Boolean Union” block, which resulted in a single implicit body.

The next stage was to create lattice structures within that cubic body. In order to do so, the first step was to define the “Unit Cell” and the “Cell map”, both of which would be used as inputs into the “Periodic Lattice” block to create the lattices. Six types of unit cells from the “Walled TPMS (WTPMS) Unit cell” block and 23 unit cells from the “Graph Unit cell” block were used to define the unit cell of the lattices. The unit cells are listed in Table 2.

After that, the “Rectangular Cell map” block was used to create a rectangular cell map within the implicit body. The important parameter of this block was cell size, which could be varied along the X, Y, and Z axes. In our paper, 20, 25, and 30 mm were used as cell sizes along three axes. Later, the “Period lattice” block was used to generate the lattices by combining the unit cells and cell maps. Here, “thickness” is a vital parameter, and we have used 2, 3, and 4 mm in that field. Lastly, the final part for a 50 × 50 × 54 mm^3^ lattice structure was created by using the “Boolean Intersect” and the “Boolean Union” blocks, where the periodic lattice and single implicit body from the earlier steps were used as inputs. Figure 1 shows a 50 × 50 × 54 mm^3^ lattice structure with a face-centered cubic foam unit cell with a cell size of 25, 25, and 30 mm along the X, Y, and Z axes with a thickness of 3 mm.

Meshing is the method of dividing a 3D model into many elements in order to accurately define its shape. In nTop, Mesh (surface mesh), Volume Mesh, and Finite Element (FE) Mesh are the three primary types of meshes. FE Mesh is a solid mesh and is used for simulation. Our objective is to convert the implicit body designed in the previous step into an FE Mesh so that simulation can be run on that body.

nTop recommends several steps that need to be followed for the conversion process that is shown in Figure 1. First, a mesh from the implicit body was created; however, meshes usually need further refinement to reduce file size, decrease element (triangle) count, and capture fine details before they can be used for simulation. “Simplify Mesh by amount” is one such method, which reduces the number of triangles on the surface mesh, depending on the amount entered. For example, an amount input of 0.5 removes half of the mesh elements. Later, the “Remesh surface” option was used to clean the defects of the parts and to consolidate meshes into fewer elements. After that, the surface mesh was converted to the solid mesh by the “volume mesh” option and, finally, “FE Volume Mesh” was used to convert the solid mesh into FE Mesh, which was used for the simulation.

### 2.3. Computational Analysis

The material used in this study was polyethylene (PE). To simulate the properties of PE, the following parameters were used in the “Isotropic Material” block as shown in Table 3 [47].

The final FE Solid model was created by combining the material block and the FE volume mesh block.

**Figure 2:**
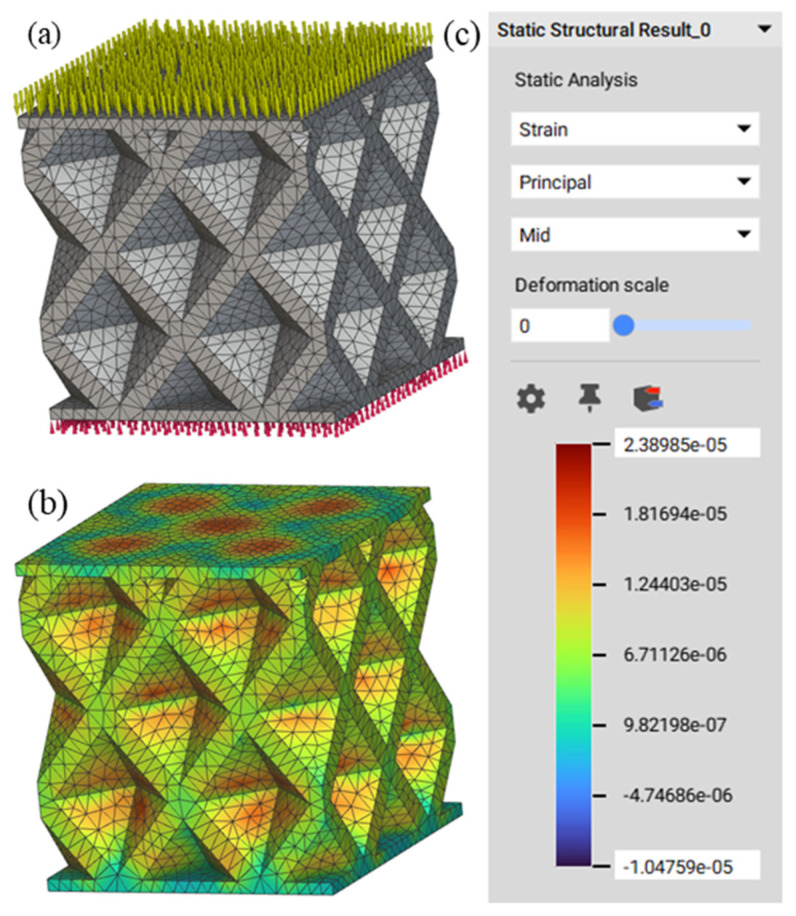
(**a**) Solid body after applying the boundary conditions; (**b**) Solid model after the simulation showing strain distribution; (**c**) Scale bar.

After that, the bottom part of the solid model was restrained (shown in red spikes in Figure 2a) and 50 N force was applied to the top part of the solid body (shown in greenish spikes in Figure 2a). The overall boundary condition is shown in Figure 2a. The static analysis block in nTop was used to run the simulations. The maximum mid-strain value found in this example was 2.38985 × 10^−5^ as shown in the following Figure 2b. Similarly, 360 simulations were conducted by varying the type of lattice, the length of the cell along the X, Y, and Z axes, and the thickness of the cell.

### 2.4. Pre-Processing: Converting and Splitting the Dataset

Every ML algorithm follows similar steps to obtain a prediction model. Once the dataset is generated, it needs to be pre-processed in order for a statistical model to tackle the real-world issue [48]. Pre-processing the dataset for ML algorithms prepares a subset of the data for training purposes. The dataset for this study had two types of inputs: lattice type was a categorical input and four numerical inputs were cell X, Y, and Z-lengths, as well as wall thickness. As a first stage in the pre-processing step, the categorical input, i.e., lattice type was converted into a numerical input using the One-Hot encoding method. In this method, the categorical input vector is transferred to the number of categories, and each training sample must be assigned only one of these inputs. For numerical inputs, the normalization of data to scale inputs in the same range would facilitate faster prediction models as well as obviate numerical overflow. The following Equation (1) was used to normalize the dataset in order to realize a standard normal distribution [48].

(1) $$x_{s}^{j}=\frac{x^{j}-\mu^{j}}{\sigma^{j}}$$

Here, $x_{s}^{j}$ is the scaled data point of input *j*; $\mu^{j}$ is the average of input *j*; and $\sigma^{j}$ is the standard deviation of input *j*. Table 4 shows a part of the converted and normalized dataset used in this study.

The next stage in the pre-processing step was splitting shuffled datasets into training and testing datasets. The purpose of the training dataset is to train the ML algorithms to establish the correlations between input and output data points; the testing dataset is used to evaluate the model developed using the training dataset [49]. In this study, for each ML algorithm, 80% and 20% of datasets were considered as training and testing datasets, dividing the entire dataset into two groups of 288 and 72 data points, respectively.

### 2.5. Training and Testing Datasets

There are two key methodologies in ML: supervised learning and unsupervised learning. In supervised learning, the algorithm is trained using labeled data and generates predictions for new, unseen data; in unsupervised learning, it independently discovers relationships among the inputs in unlabeled data [50]. In this paper, since the five lattice parameters were varied to read associated strains as the output, the problem was considered supervised learning. Additionally, because the strain output is numerical, the applied ML algorithms must follow the rules associated with regression problems. The following sections describe the five ML algorithms that were evaluated in this study.

The ML algorithms were run on a system configuration consisting of an 11th Gen Intel^®^ Core™ i5 processor with four cores and a clock speed of 2.40 GHz, as well as 8.00 GB of RAM, running on the Microsoft Windows 11 Home operating system. The ML algorithms were implemented using the Python programming language version 3.9.13 in the Jupyter Notebook environment, utilizing the Sklearn, Tensorflow, and Keras libraries. The hyperparameters for ANN were tuned using the gridsearchCV module in the Jupyter Notebook environment.

#### 2.5.1. Linear Regression

Linear regression (LR) is a widely used statistical technique that models the correlation between specific inputs and numerical outputs. In supervised machine learning, LR models excel at discovering the optimal linear relationship between the predictors and the response variable, offering ease of interpretation, making them a preferred choice when a linear relationship is suspected or when a straightforward and computationally efficient regression model is sought [49]. If there are N samples with D inputs, and if the inputs are expressed as $x_{i}^{j}$, where *i* is the number of sample *i* = 1, …, N, *j* is the number of inputs *j* = 1, …, D, the output true or target values are $y_{i}$. The LR model utilizes a function expressed by Equation (2) [48].

(2) $$f_{w,b}\left(X\right)=wX+\varepsilon$$

where $f_{w,b}\left(X\right)$ is the predictor matrix, *X* is the D-dimensional vector of inputs, *w* is the D-dimensional vector of coefficient, and *ε* is the total error. The aim is to find $f_{w,b}\left(X\right)$ by adjusting *w* and minimizing *ε* [48].

Squared error loss is a particular loss function that measures the penalty for mismatched predictions, which is commonly used in ML algorithms. In model-based learning algorithms, the objective is to attempt to minimize the cost function to find the best prediction model. The cost function for the LR model is determined by the average loss, which is the average of all penalties obtained by using the model on the training data. Therefore, the less the *ε* in Equation (2), the less the error that the prediction model has. There are various cost functions for evaluating the model learned by the algorithms that will be described in Section 3.

#### 2.5.2. Polynomial Regression

Using a straight line to represent the relationship between the inputs and any outputs is insufficient for non-linear relationships. In such cases, exploring non-linear relationships between variables can result in a better model [51]. Polynomial regression (PR) is a useful method for capturing non-linear patterns in data by incorporating polynomial terms, thereby extending the capabilities of linear regression. The PR model is commonly employed to incorporate higher-order terms of the input parameters (independent variables), thereby facilitating a more comprehensive examination of non-linear associations within the dataset [52]. Hence, PR models should be able to better capture the true correlation between input/output parameters in our dataset. This model can be expressed by the following Equation (3).

(3) $$f_{w,b}\left(X\right)=w_{0}+w_{1}X+w_{2}X^{2}+w_{3}X^{3}+\cdots +w_{p}X^{p}+\varepsilon$$

Increasing the degree of the polynomials in the equation can make the model more complex which, in turn, can lead to overfitting [51]. Overfitting occurs when the model is trained on a particular dataset and shows high accuracy, but performs poorly when tested on a new dataset [51]. To avoid overfitting, it is important to be cautious when using this ML algorithm; however, this paper did not exhibit overfitting because of the small errors for both training and testing datasets. In this study, the model showed the lowest error for *p* = 1 degree of the PR model.

#### 2.5.3. Decision Tree

The Decision Tree (DT) ML model can map inputs to output. The tree predicts the label of a data point by following a path from the root node to a leaf node. The root node, situated at the highest level of the decision tree, serves as the initial point of data division. This term refers to the complete set of data that is utilized for training purposes. A leaf node is the terminal or final node in a decision tree; it represents a specific numerical value in regression problems, which has been assigned to the data instance that reaches this node. At each node along the path, the tree uses a splitting rule to decide which child node to follow. The splitting rule typically involves checking the value of a particular input of the samples or applying a set of predefined rules [53].

DT is used for both classification and regression problems. In this study, DT for regression, commonly known as a regression tree, was applied. This is used for predicting continuous target variables. The process of building a regression tree involves binary recursive partitioning, which involves iteratively splitting data into partitions based on a selected splitting rule that minimizes the sum of squared deviations from the mean in the resulting subgroups. Initially, all training set records are grouped into the same partition; the algorithm then selects the best split for each partition based on the minimum sum of squared deviations [48].

DT is widely recognized for its versatility and proficiency in managing any non-linear and non-monotonic relationships present in data. Consequently, it is highly regarded as a valuable tool for the identification of essential features. These trees employ sophisticated split decisions and suitable stopping criteria, facilitating efficient decision making, event forecasting, and identification of consequences [54]. In this study, the maximum depth of trees considered was 35; however, the best tree with the minimum mean squared error (MSE) was found with a maximum depth of 9.

#### 2.5.4. Random Forest

Random Forest (RF) was selected for this study primarily based on its robust predictive abilities. It exhibits a high degree of versatility as it can be effectively employed in both regression and classification tasks, rendering it a viable option for a wide range of data-analysis purposes. The RF algorithm is an ensemble technique that combines multiple decision trees, consolidates their predictions, and reduces overfitting. This approach provides several advantages, including enhanced robustness, resilience to outliers, and improved generalization capabilities compared to individual decision trees [55].

RF prevents correlation among trees by preventing strong predictors to split data points in multiple trees. In other words, the algorithm creates trees that are as independent as possible from each other. This is achieved by randomly selecting subsets of inputs and samples for each tree so that each tree learns to make predictions based on different combinations of inputs and samples. By doing so, the trees become less correlated and produce more diverse predictions, which can improve the accuracy and robustness of the RF model [48].

RF prediction considers individual trees that produce models with low variance and reduced risk of overfitting. This technique is widely used in ensemble learning [48]. In this research, an investigation was conducted to find out the maximum depth of trees for the RF algorithm. A depth of nine was found to be optimal for achieving the highest performance based on evaluation metrics such as MSE, mean absolute error (MAE), and root mean square error (RMSE). These findings suggest that the choice of hyperparameters, such as the maximum depth of trees, can significantly impact the effectiveness of the RF algorithm.

#### 2.5.5. Artificial Neural Network

Artificial Neural Network (ANN) is a computational model inspired by the structure of neural networks in the brain. The network consists of a large number of interconnected computing devices called neurons, which carry out complex computations. A neural network is represented as a directed graph with neurons as nodes and edges as links between them. Neurons receive inputs from connected neurons and produce outputs that are passed on to other connected neurons [53].

A feedforward neural network, also known as a multi-layer perceptron, in which information flows in one direction, from the input layer to the output layer, is a stack of several hidden layers, with the final output being only one layer. Each neuron of each layer is associated with an activation function; the activation function of the last layer, which has only one neuron, determines the type of model. Linear activation function results in a regression model, which is used to predict numerical values; a logistic activation function creates a binary classification model, which is used to sort data into two classes. The type of model is selected based on the problem definition [48,53].

The ANN possesses significant efficacy in tackling intricate engineering problems due to its capacity to represent intricate, non-linear associations within data. With recent advancements in computing and algorithms, ANNs have been extensively employed to predict system behavior. These networks have proven to be highly effective, particularly in scenarios involving non-linear behavior. These computational systems are influenced by the biological neural networks found in the human brain, which consist of artificial neurons that receive and process input signals using mathematical operations. ANNs exhibit a high level of suitability for tasks that involve extensive datasets and the automatic acquisition of feature representations, and are highly adaptable for diverse applications [56,57].

This study employed the Grid Search method and Cross-Validation technique to optimize the hyperparameters of ANN. The utilization of this cross-validation technique can yield a more dependable estimation of a model’s performance compared to a solitary train-test split. Cross-validation can be employed to identify overfitting by evaluating the model on different subsets of the data [56]. The training dataset was subjected to a cross-validation approach using a 5-fold method.

The hyperparameters that were taken into consideration for each ML algorithm in this study, along with their respective values, are provided in Table 5. The consideration of the number of hidden layers was also taken into account for the purpose of tuning; nevertheless, the findings of this study indicate that the performance of the model did not exhibit a substantial enhancement when the number of hidden layers was increased, likely due to the limited size of the dataset. It is worth noting that this study exclusively employed a single hidden layer in its analysis.

Learning rate is a pivotal hyperparameter in a predictive model and should be prioritized for tuning. This factor plays a crucial role in the determination of the magnitude of the optimizer’s increments when modifying the weights of the network during the training process. The magnitude of weight updates, and the convergence rate of the network to the optimal solution, are influenced by the learning rate [58]. Furthermore, the remaining hyperparameters to be optimized, in a sequential fashion, encompassed the activation function, batch size, epochs, and the number of neurons within the hidden layer.

The results of hyperparameter optimization for ANN applied in the prediction of strain in AM are displayed in Table 6. This table presents the optimal values of the pre-determined hyperparameters that were evaluated in this study, leading to the selection of the most effective predictive model. The results emphasize the significance of precise hyperparameter selection and tuning in ANN models in order to attain optimal performance.

**Figure 3:**
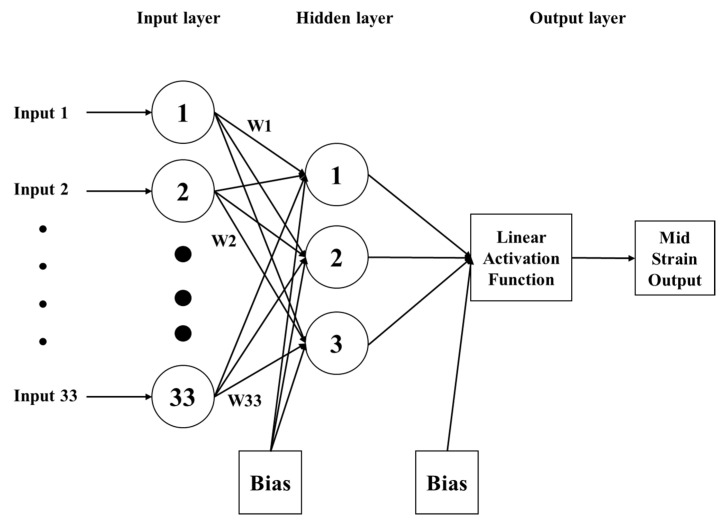
Schematic of best model of ANN for this study.

In this paper, the hyperparameters of ANN were fine-tuned; these included the number of layers, number of neurons, activation function for each layer, learning rate, batch size, and number of epochs (i.e., one full training cycle). Through experimentation, it was determined that the optimal configuration for these hyperparameters was as follows: 1 hidden layer with 3 neurons (which are shown in the hidden layer in Figure 3); a linear activation function for the hidden layer; a learning rate of 0.001; a batch size of 2; and 200 epochs, as shown in Figure 3. In addition, 33 neurons in the input layer is demonstrating the number of inputs which were shown in Table 1. These findings highlight the importance of carefully selecting and tuning the hyperparameters of ANN models to achieve optimal performance.

## 3. Error Metrics for ML Models

In statistical analysis, it is commonplace to use measures of error or accuracy to evaluate the performance of a predictive model. This study utilized three error metrics to assess the performance of a regression model: mean squared error (MSE), root mean squared error (RMSE), and mean absolute error (MAE).

MSE is a widely used measure of error in regression analysis; it calculates the average of the squared differences between predicted and actual values. The formula for MSE is provided in Equation (4):

(4) $$MSE=\frac{1}{n}\sum_{i=1}^{n}{\left(y_{i}-{\hat{y}}_{i}\right)}^{2}$$

where *n* is the sample size, *y_i_* is the actual value, and *ŷ_i_* is the predicted value [53].

RMSE is the square root of MSE. A lower RMSE indicates a better fit between the predicted and actual values, meaning that the model has a higher degree of accuracy in estimating the dependent variable; however, the interpretation of the RMSE also depends on the scale of the dependent variable [59]. The formula for RMSE is expressed in Equation (5):

(5) $$RMSE=\sqrt{\frac{1}{n}\sum_{i=1}^{n}{\left(y_{i}-{\hat{y}}_{i}\right)}^{2}}\;$$

MAE is another commonly used measure of error in regression analysis. Similar to RMSE, the interpretation of the MAE also depends on the scale of the dependent variable. It measures the average of the absolute differences between the predicted and actual values [60]. The formula for MAE is represented by Equation (6):

(6) $$MAE=\frac{1}{n}\sum_{i=1}^{n}\left|y_{i}-{\hat{y}}_{i}\right|$$

## 4. Evaluation of ML Models

For analyzing and interpreting results, it is crucial to report the values of these error metrics to demonstrate the performance of a model. Selecting which measures to report depends on the research question, the type of data, and the specific analysis conducted. The results of training five ML algorithms concerning their error metrics for the training and testing phases are shown in Table 7.

The results presented in Table 7 indicate that the prediction errors for the metrics MSE, RMSE, and MAE are remarkably low during both the training and testing phases for all ML models. This leads to the creation of a dependable and credible model for each of the ML algorithms used. Furthermore, the LR and PR algorithms exhibit similar accuracy levels. This can be attributed to the fact that the degree of order obtained with the PR algorithm is 1, which implies that the optimal model for our dataset under the PR algorithm follows a linear model, similar to that of the LR algorithm.

**Figure 4:**
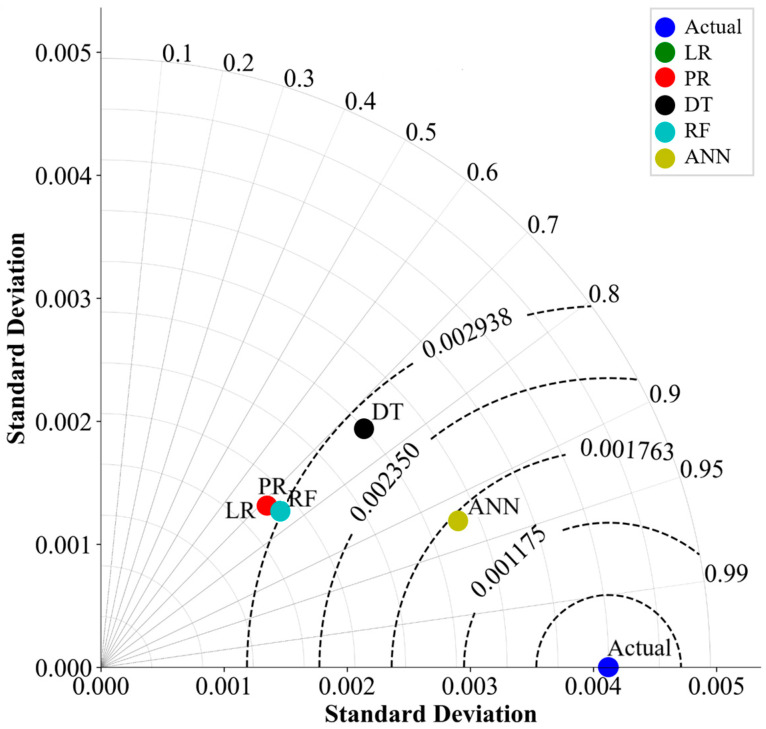
Taylor Diagram.

The Taylor diagram is a visual aid that is used to compare models or observations to a reference dataset in terms of correlation, variability, and bias on a single chart [61]. The diagram is constructed using a polar coordinate system, with the actual dataset depicted as a point at the center. Each model is plotted as a point on the diagram, with the distance from the origin representing its correlation with the actual dataset and the angular position representing the ratio of standard deviations between the model and the actual dataset. The distance between a model and an actual dataset is visualized by arcs using RMSE. The closer a point is to the reference point, the better the model’s performance [61]. The Taylor diagram in Figure 4. illustrates the results of the comparison between the five ML algorithms in this study. The diagram plots the actual point, which represents the standard deviation of the test dataset, and each algorithm is represented by a point in the plot. The algorithm whose point is closest to the actual point on the diagram is ANN. As this plot demonstrates, ANN’s correlation is 0.93, which is followed by DT, which is 0.74. This indicates that the ANN model has a high correlation with the actual data in this research, and its RMSE is close to zero. Therefore, the Taylor diagram suggests that the ANN algorithm outperformed the rest of the ML algorithms in this study. As mentioned before, LR and PR provide identical outcomes, so their overlap in the Taylor diagram is also evident.

Nevertheless, while the correlation of ANN may be satisfactory, there are various approaches that can enhance the predictive capabilities of ML models. This study employed hyperparameter tuning as a means to reduce overfitting and enhance the accuracy of the models; however, an additional method to enhance prediction accuracy is to collect more data. The utilization of a broader and more representative sample in training models helps to alleviate the issue of overfitting. Furthermore, the process of identifying and selecting the features that are most relevant has the potential to enhance the precision of the prediction. The simplification of the model and enhancement of accuracy can be achieved by eliminating irrelevant or redundant features [62].

This research also utilized the relative error box plot to evaluate the accuracy of ML algorithms in predicting a model. This plot measures the percentage difference between the predicted value and true value. This is an important tool for assessing the precision of a model’s predictions. It can be used to compare different ML algorithms for a given dataset [63].

**Figure 5:**
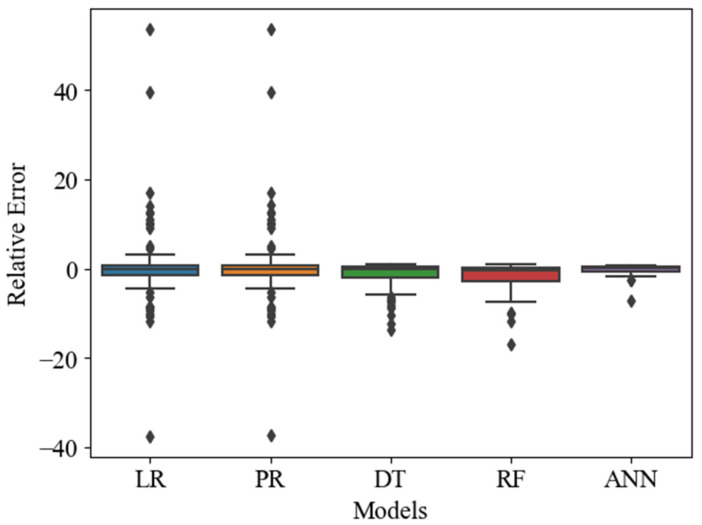
Relative error box plot.

In this study, the box plot of relative error for each ML algorithm is presented in Figure 5, with the results indicating that the median value for the ANN algorithm was the lowest in comparison with other ML models. The ANN algorithm also exhibited a smaller interquartile range, indicating that its errors were more consistent across different data points. Conversely, the LR and PR algorithms had several error values falling outside of the box which are shown in diamond shape, indicating difficulties in accurately predicting certain types of data points. Additionally, the narrower box plot of the ANN algorithm suggests a more tightly clustered distribution compared to other algorithms.

**Figure 6:**
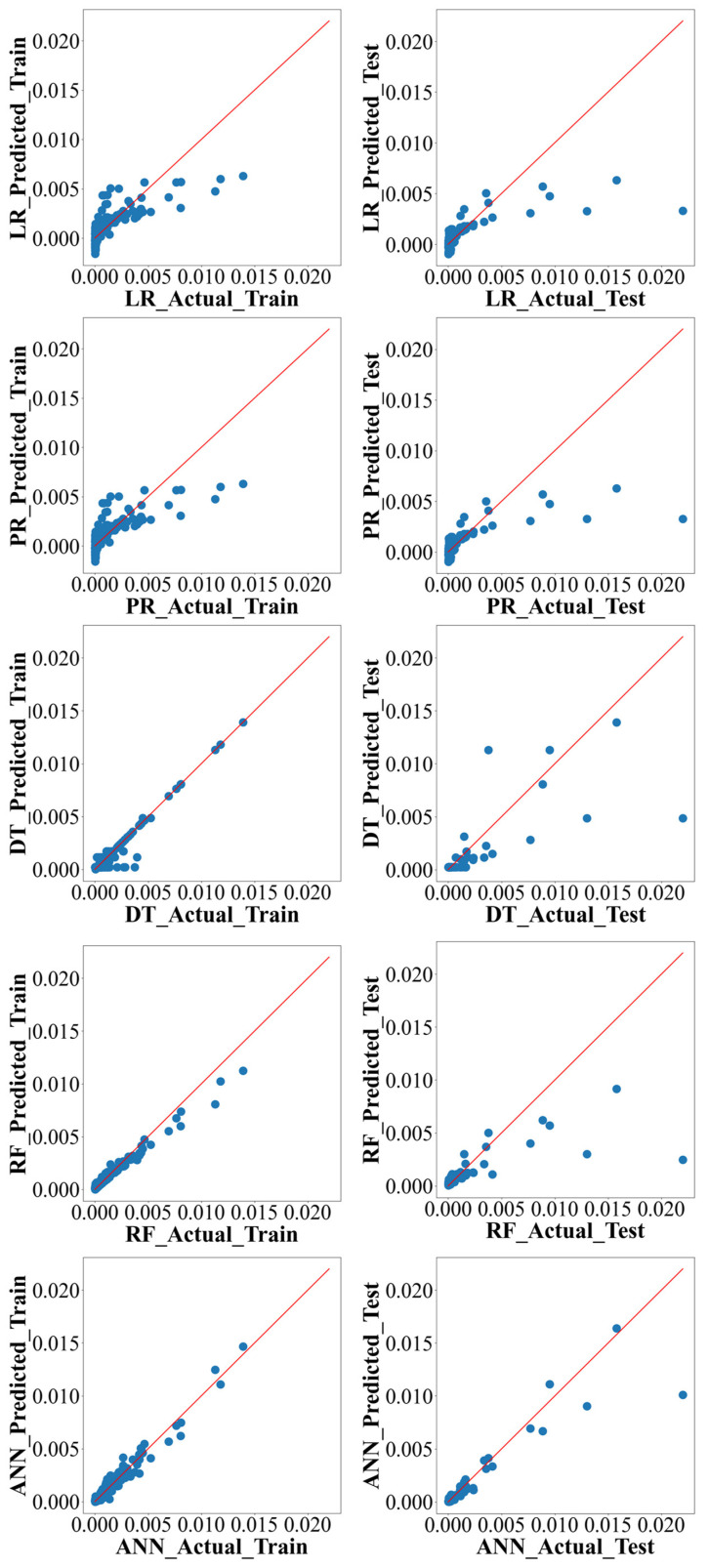
Comparison between predicted vs. actual values of training and test models.

Moreover, Figure 6 illustrates a comparative analysis of ML algorithms, focusing on their performance in predicting actual values vs. predicted values throughout the training and testing phases. It shows the superior performance of ANN compared to other algorithms. It is worth mentioning that the ANN model consistently demonstrates the highest level of agreement between observed and forecasted values, thus confirming its effectiveness as the preferred algorithm for precise predictions within this particular framework, other than the ML algorithms for this research. The presented visual evidence serves to emphasize the importance of this study’s findings and the potential implications of employing ANN in practical scenarios that require accurate prediction.

**Figure 7:**
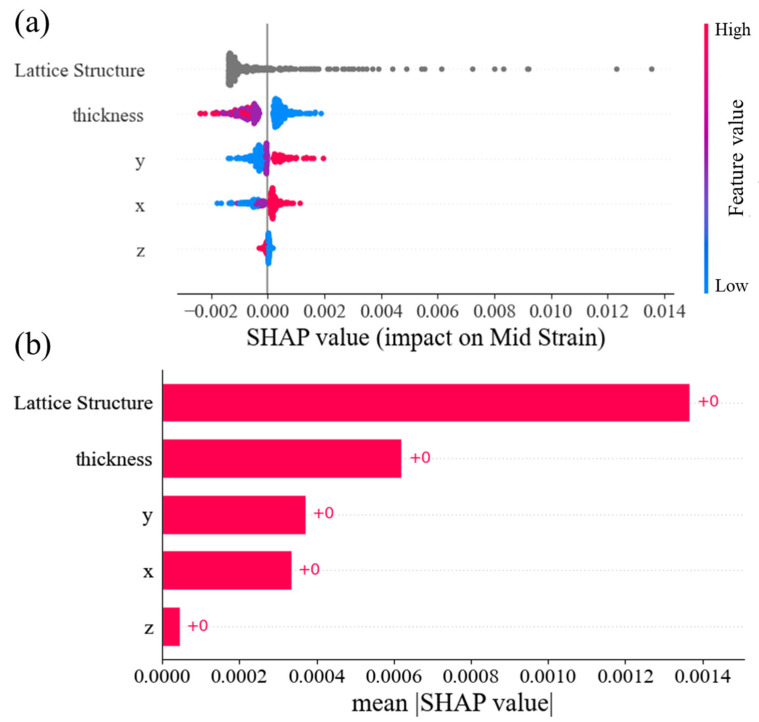
The importance of each feature using SHAP with (**a**) beeswarm plot and (**b**) plot bar of absolute SHAP values.

The SHapley Additive exPlanations (SHAP) method, initially proposed by Lundberg and Lee [64], was also utilized in this study to determine the individual contributions of each feature. This methodology, based on co-operative game theory, improves the clarity and comprehensibility of ML models [65]. In order to evaluate the importance of features within the entire dataset, this study employed a bee swarm plot. As depicted in Figure 7a, the variables have been organized based on their global feature importance, with the most significant variables positioned at the top and the least significant variables positioned at the end. With the given dataset and the best ANN model in this study, it was observed that the lattice structure feature had a significant positive effect when its values were high, while its impact was relatively minor and negative when the values were low. The influence of the feature’s Z-axis on strain predictions was found to be minimal, regardless of whether its values were high or low. The reason for showing the lattice structure feature with a different color than other features in Figure 7. is that this feature is a categorical feature while others are numerical.

Furthermore, the bar plot depicted in Figure 7b illustrates that the order of features is determined by their absolute SHAP values, regardless of their impact on predictions, be they positive or negative. In conclusion, the most important features for strain in this study are lattice structure, thickness, Y, X, and Z, respectively.

## 5. Conclusions

In conclusion, this study successfully developed a strain prediction model for designing lattice structures for AM-processed ordered foam material ML algorithms. First, a dataset of 360 data points was generated from 29 types of lattice structures, by varying the thickness and cell size of those structures along the X, Y, and Z axes. Then, by utilizing that dataset and employing supervised learning methods in ML with regression models, the study was able to accurately predict the mechanical deformation of the lattice structures, namely, strain. The study compared the performance of five ML algorithms, including Linear Regression, Polynomial Regression, Decision Tree, Random Forest, and Artificial Neural Network, and found that the ANN algorithm outperformed the others. Evaluation metrics such as mean squared error, root mean squared error, and mean absolute error, showed remarkably low prediction errors during both the training and testing phases, indicating a dependable and credible model for each of the ML algorithms used. The visualization of the system’s output through the Taylor diagram and relative error box plot, and comparison between the actual and predicted values of training and testing phases, further confirmed the superiority of the ANN algorithm; moreover, this study used the SHAP method to evaluate feature importance across the dataset and its contribution to the predictions, which showed that lattice structure had a significant positive effect when values were high, while the Z-axis had minimal influence. Overall, the results of this study have important implications for the development of accurate and reliable strain prediction models for lattice structures in AM, which could contribute to improving the quality and efficiency of AM processes in various industries.

## Figures and Tables

**Table 1:** Lattice parameters and the levels used in designing lattice structures.

Lattice Parameters	Number of Levels	Point Levels
Lattice Types	29	Six (6) WTPMS unit cells and twenty-three (23) graph unit cells
Cell X-length	3	20 mm, 25 mm, and 30 mm
Cell Y-length	3	20 mm, 25 mm, and 30 mm
Cell Z-length	3	20 mm, 25 mm, and 30 mm
Wall Thickness	3	2 mm, 3 mm, and 4 mm

**Table 2:** Twenty-Nine (29) different types of lattice unit cells from nTop’s library.

WTPMS Unit Cell	Graph Unit Cell			
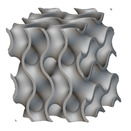	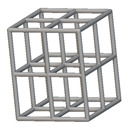	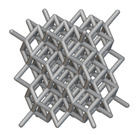	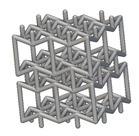	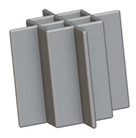
Gyroid	Simple Cubic	Fluorite	Re-entrant	Square Honeycomb Rotated
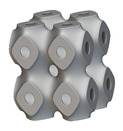	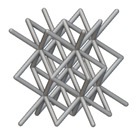	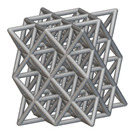	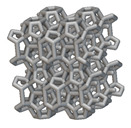	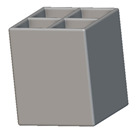
Schwarz	Body-Centered Cubic	Octate	Weaire-Phelan	Square Honeycomb
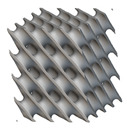	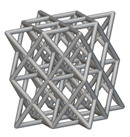	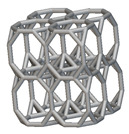	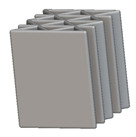	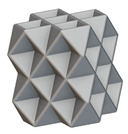
Diamond—WTPMS	Face Centered Cubic	Truncated Cube	Triangular Honeycomb	Face Centered Cubic Foam
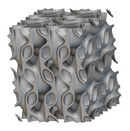	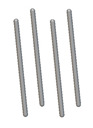	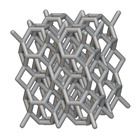	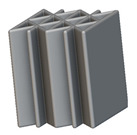	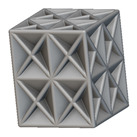
Lidinoid	Column	Truncated Octahedron	Triangular Honeycomb Rotated	Body-Centered Cubic Foam
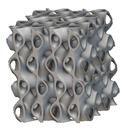	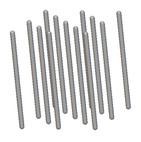	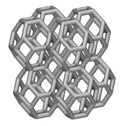	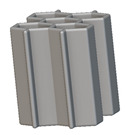	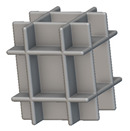
Split P	Columns	Kelvin Cell	Hexagonal Honeycomb	Simple Cubic Foam
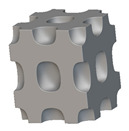	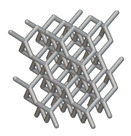	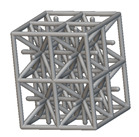	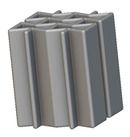	
Neovius	Diamond—Graph	Isotruss	Re-entrant Honeycomb	

**Table 3:** Material properties of polyethylene (PE).

Properties	Value
Young’s Modulus	1.1 × 109 Pa
Poisson’s ratio	0.42
Density	0.00095 gm/mm^3^

**Table 4:** The converted and normalized dataset.

	BC	BF	CL	CS	DG	DW	FC	FF	FL	GD	…	SZ	TC	TH	TO	TR	WP	X	Y	Z	Thickness
**0**	0	0	0	0	0	0	0	0	0	1	…	0	0	0	0	0	0	−1.523839	−0.746602	−0.861621	−0.856103
**1**	0	0	0	0	0	0	0	0	0	0	…	1	0	0	0	0	0	−1.523839	−0.746602	−0.861621	−0.856103
**2**	0	0	0	0	0	1	0	0	0	0	…	0	0	0	0	0	0	−1.523839	−0.746602	−0.861621	−0.856103
**3**	0	0	0	0	0	0	0	0	0	0	…	0	0	0	0	0	0	−1.523839	−0.746602	−0.861621	−0.856103
**4**	0	0	0	0	0	0	0	0	0	0	…	0	0	0	0	0	0	−1.523839	−0.746602	−0.861621	−0.856103
**…**	…	…	…	…	…	…	…	…	…	…	…	…	…	…	…	…	…	…	…	…	…
**355**	0	0	0	0	0	0	0	0	0	0	…	0	0	1	0	0	0	−1.523839	−0.746602	1.299937	0.551189
**356**	0	0	0	0	0	0	0	0	0	0	…	0	0	0	0	0	0	−0.376178	−0.746602	−0.861621	−0.856103
**357**	0	0	0	0	0	0	0	0	0	0	…	0	0	0	0	1	0	0.771484	0.427095	1.299937	1.958481
**358**	0	0	0	0	0	0	1	0	0	0	…	0	0	0	0	0	0	−0.376178	0.427095	−0.861621	1.958481
**359**	0	0	0	0	0	0	0	0	0	0	…	0	0	0	0	0	0	−0.376178	0.427095	1.299937	1.958481

**Table 5:** Tuned hyperparameters for ANN and their values.

Hyperparameters	Values
Learning rate	0.001, 0.005, 0.01, 0.02, 0.04, 0.05
Activation function for a hidden layer	softmax, softplus, softsign, relu, tanh, sigmoid, hard_sigmoid, linear
Number of batches	1, 2, 3, 4, 5, 6, 7
Number of epochs	200, 300, 400, 500
Number of neurons for a hidden layer	3, 4, 5, 6, 10

**Table 6:** Values of the best model for hyperparameters of ANN.

Hyperparameters	Value
Learning rate	0.001
Activation function for a hidden layer	linear
Number of batches	2
Number of epochs	200
Number of neurons for a hidden layer	3

**Table 7:** The results of evaluation metrics.

	Training Results			Testing Results		
	MSE	RMSE	MAE	MSE	RMSE	MAE
LR	1.264 × 10^−6^	1.124 × 10^−3^	6.677 × 10^−4^	8.624 × 10^−6^	2.937 × 10^−3^	1.191 × 10^−3^
PR	1.264 × 10^−6^	1.124 × 10^−3^	6.677 × 10^−4^	8.624 × 10^−6^	2.937 × 10^−3^	1.191 × 10^−3^
DT	2.007 × 10^−7^	4.480 × 10^−4^	2.262 × 10^−4^	6.659 × 10^−6^	2.580 × 10^−3^	9.644 × 10^−4^
RF	1.467 × 10^−7^	3.830 × 10^−4^	1.761 × 10^−4^	8.103 × 10^−6^	2.847 × 10^−3^	9.873 × 10^−4^
ANN	1.133 × 10^−7^	3.366 × 10^−4^	1.925 × 10^−4^	2.453 × 10^−6^	1.566 × 10^−3^	4.653 × 10^−6^

## Data Availability

Data beyond results discussed in this article can be made available upon request to the corresponding author.
